# Potentials of plant-based substance to inhabit and probable cure for the COVID-19

**DOI:** 10.3906/biy-2005-114

**Published:** 2020-06-21

**Authors:** Israt JAHAN, Ahmet ONAY

**Affiliations:** 1 Department of Bioengineering, Faculty of Chemical and Metallurgical Engineering, Yıldız Technical University, İstanbul Turkey; 2 Department of Biology, Faculty of Science, Dicle University, Diyarbakır Turkey

**Keywords:** COVID 19, coronaviruses, MERS-CoV, SARS-CoV, antiviral plants, emodin, HCoV-229E, lycorine

## Abstract

COVID-19 has been the most devastating pandemic in human history. Despite the highest scientific efforts and investments, a reliable and certified medication has yet to be developed regarding to immune or cure this virus. However, while synthetic medications are gaining the focus of attentions, it appears from a significant number of recent studies that plant-based substances could also be potential candidates for developing effective and secure remedies against this novel disease. Citing such recent works, this review primarily demonstrates the antiviral potentials of medicinal plants for inhibiting human coronaviruses. It also shows the importance of antiviral plants substances, particularly in the development of a broad spectrum medication for coronaviruses including SARS-CoV-2 responsible for COVID-19.

## 1. Introduction 

Viral diseases have always been a distressing threat for main kind, and many novel viral infections are reported constantly throughout the word with severe health issues (Zhang et al., 2020a). COVID-19 is an infectious viral disease caused by a new type of contagious and pathogenic human severe acute respiratory syndrome coronavirus-2 (SARS-CoV-2) (Lai et al., 2020). This viral infection has turned into a pandemic, which are affecting world’s population severely. Until 1:31 pm of 29 May 2020, the World Health Organization (WHO) reported that the total number of confirmed COVID-19 cases all over the world except Antarctica is 5,701,337, including 357,688 deaths; while in Turkey, total number of positive cases of COVID-19 reached to 160,979 with 4,461 deaths^1^1WHO (2020). Coronavirus Disease (COVID-19) Dashboard [online]. Website https://covid19.who.int/ [accessed 29 May 2020].. 

This ongoing pandemic is getting worse day by day, and no approved medication or vaccine has been established so far to treat COVID-19 patients (Suryanarayana and Banavath, 2020). Hence, an extensive research is being carried out to develop the effective treatment for virus-infected patients as well as determine the source, structure, infection mechanism of many coronaviruses (CoV) of zoonotic origin including SARS-CoV-2 (Ul Qamar et al., 2020). However, it is really difficult to develop a broad spectrum antiviral medications since viruses can mutate their genetic materials easily which helps them to become resistant to any available drugs (Oyston and Robinson, 2012). Albeit an effective vaccine is the certain version of the viral treatment, development of a fully potential safe to use vaccine as well as reaching it to the market level is time consuming as it must meet several steps before getting approval (Oyston and Robinson, 2012). In addition, synthetically developed antiviral medications sometimes possess inauspicious side effects, which could cause significant health problem (Ghildiyal et al., 2020). Therefore, plant-based herbal medicine with effective antiviral potential can open new opportunities by minimizing these side effects (Ghildiyal et al., 2020). 

Since the prehistoric times, people from different parts of the world have been utilizing plant for manufacturing herbal medicine to control infectious diseases, which might also possess anti-HCoV active extracts or compounds (Andrighetti-Fröhner et al., 2005; Yang et al., 2020). Medicinal plants are boosted with diverse secondary metabolites; some of them can interrupt viral protein and enzyme activities by binding with them, and prevent viral penetration, replication into the host cells (Li and Peng, 2013; Arbab et al., 2017; Akram et al., 2018; Dhama et al., 2018). Numerous studies have been confirmed the bioactive natural compounds which could be potential candidates treating the novel SARS-CoV-2 due to their significant antiviral activity (ul Qamar et al., 2020; Zhang et al., 2020a). Moreover, traditional Chinese medicines (TCM) have been very popular as supplementary therapeutic medication, and might be able to demonstrate significant positive response in preventing SARS-CoV-2. 

Presenting the origin, structure, transmission and infection mechanisms, this review primarily attempts to present a glimpse of the antiviral potentials of medicinal plants for inhibiting human coronaviruses. It also emphasizes the applications of plant-based TCM regarding to SARS coronaviruses. Besides, it shows the importance of antiviral phytochemicals of different plants, could be considerable for developing of a broad spectrum medication against the virulence effects of any types of coronaviruses including SARS-CoV-2. 

## 2. Methodology 

A literature search was conducted in the PubMed, Google Scholar and Web of Science in order to find the most recent published articles related to the keywords such as “coronavirus”, “novel coronavirus”, “CoV”, “COVID-19”, “SARS-CoV-2”, “antiviral plants”, “antiviral compounds”, “phytochemicals”, “herbal drugs”, and “Traditional Chinese Medicine” until 27 May 2020. No language restriction was imposed in this article. Some of required information was searched using following websites: National Health Commission of the People’s Republic of China (http://www.nhc.gov.cn/) and the WHO (https://www.who.int/). A brief description about coronaviruses, their origin, transmission and infection mechanism in host cell were given in the review. Significantly important phytochemicals, especially secondary metabolites with antiviral potential were classified and discussed properly by providing relevant figure. A partial list of significant phytochemicals with antiviral properties for preventing human coronaviruses was presented by a table. Moreover, this study represented an overall review about the in vitro or in vivo applications of plant-based substances on SARS-CoV-2, SARS-CoV and other human coronaviruses. 

## 3. Findings and discussion 

### 3.1. Coronavirus: origin, transmission and infection mechanism in humans 

Coronaviruses are considered as highly pathogenic and capable of causing diseases among birds and mammals (Woo et al., 2010). They are assembled into the family Coronaviridae which belongs to the order Nidovirales. This virus group is further divided into 4 genera, i.e. alphacoronaviruses (α-CoV), betacoronaviruses (β-CoV), gammacoronaviruses (γ-CoV), and deltacoronaviruses (δ-CoV) (Woo et al., 2010). Alphacoronaviruses and betacoronaviruses have the tendency to infect mammals, whereas gammacoronaviruses and deltacoronaviruses are capable of infecting birds. Two betacoronaviruses (HCoV-HKU1 and HCoV-OC43) and 2 alphacoronaviruses (HCoV-229E and HCoV-NL63) are well-known and recognized as low pathogenic virus for human, and responsible for mild respiratory symptoms, which is identical to seasonal flue (Woo et al., 2007). However, the betacoronaviruses (β-CoV) have earned the greatest concern among all due to their clinical importance regarding the capability of causing serious illness in human race. Middle East respiratory syndrome-related coronavirus (MERS-CoV) and severe acute respiratory syndrome coronavirus (SARS-CoV, SARS-CoV-2) are the most discussed betacoronaviruses of zoonotic origin because of their extreme capacity of developing fatal respiratory tract infections in human (Lu et al., 2020). 

**Figure 1:**
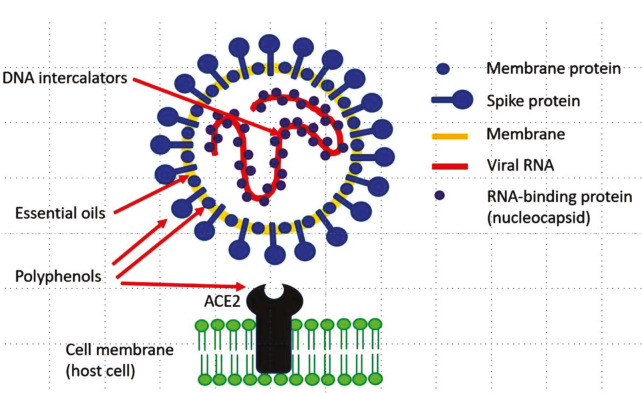
Illustration of the minimal composition of a severe acute respiratory syndrome coronavirus 2 (SARS-CoV-2) (Wink, 2020).

Coronaviruses are large, sphere-shaped, single-stranded RNA virus of approximately 125 nm in diameter (Fan et al., 2019). Their genomic RNA is the largest genome among all RNA viruses (size range between 26.2 and 31.7 kb, positive sense) (Fan et al., 2019). This single strand RNA (+ sense) is enclosed by an enveloped structured protein shell/ capsid protein of helical symmetry (Figure 1). This enclosed helical structure of genomic material and protein is known as nucleocapsid, which is further enfolded by an icosahedral protein shell (De Groot et al., 2011). Single-strand RNA and enfolded protein shells are again surrounded by a bulbous surface lipid bilayer, in which different structural protein i.e. spike (S), envelope (E), and membrane (M) are anchored at the ratio of 20:1:300 (Figure 1) (Lai and Cavanagh, 1997). A coronavirus particle can have about 74 surface spikes on average. The nature of the tissue tropism of a host cell as well as the binding pattern of viral spike protein and its suitable host cell receptor determine the species range of a virus and its infectivity (Neuman et al., 2011). In together, nucleocapsid, lipid bilayer and membrane proteins can provide protection when the virus is outside without its carrier or a suitable host (Neuman et al., 2011). 

Considering the findings of evolutionary investigations and virus genome sequencing, it has found that warm-blooded flying vertebrates are the most suitable and impeccable natural host and reservoir for alphacoronaviruses and betacoronaviruses (Yin and Wunderink, 2018). For instance, in case of SARS-CoV-2, bat is believed to be the highly probable primary source of this virus which may jumped to unknown intermediate hosts, and then transmitted and infected human, and consequently caused the novel COVID-19. This is due to the fact that bat CoV RaTG13 virus and SARS-CoV-2 share 96.2% of identical genomic sequence which suggested that both viruses might came from the same ancestor (Zhou et al., 2020). 

Like any other coronaviruses, the main course of the transmission of SARS coronaviruses from human to human is by direct or indirect contact with infected carriers. Besides in a short range transmission, these viruses can spread by respiratory droplets (Moriyama et al., 2020). It is also evidenced that for long-range transmission, coronaviruses are able to transmit through aerosol (Moriyama et al., 2020). After shedding into the environment from infected host, SARS-CoV-2 and other coronaviruses can float to the ground and any other suitable surface through droplets, which help them to stay alive for a certain period of time, and infect again once come to the contact with new host (Lai et al., 2020). 

Virus-host interactions are the most significant factors that influence viral entry and replication in host cells. Human epithelial cells are mostly targeted region by coronaviruses at the time of infection. SARS-related coronavirus enter into the human body through an aerosol route and attached with the epithelial cells of the respiratory tract (Ge et al., 2013). The S-proteins of the virus and their receptor of SARS-CoV-2 and other human coronaviruses play the main role to penetrate into the epithelial cells of human lung (Letko et al., 2020). For the attachment, spike protein receptor binding domain (RBD) of human SARS coronaviruses binds with angiotensin-converting enzyme 2 (hACE2) available in the outer cell membrane of human lung (Figure 1) (Letko et al., 2020). On the other hand, binding domain (RBD) of MERS-CoV interacts with an extracellular type-II transmembrane glycoprotein called dipeptidyl peptidase-4 (DPP-4) (Mubarak et al., 2019). 

**Figure 2:**
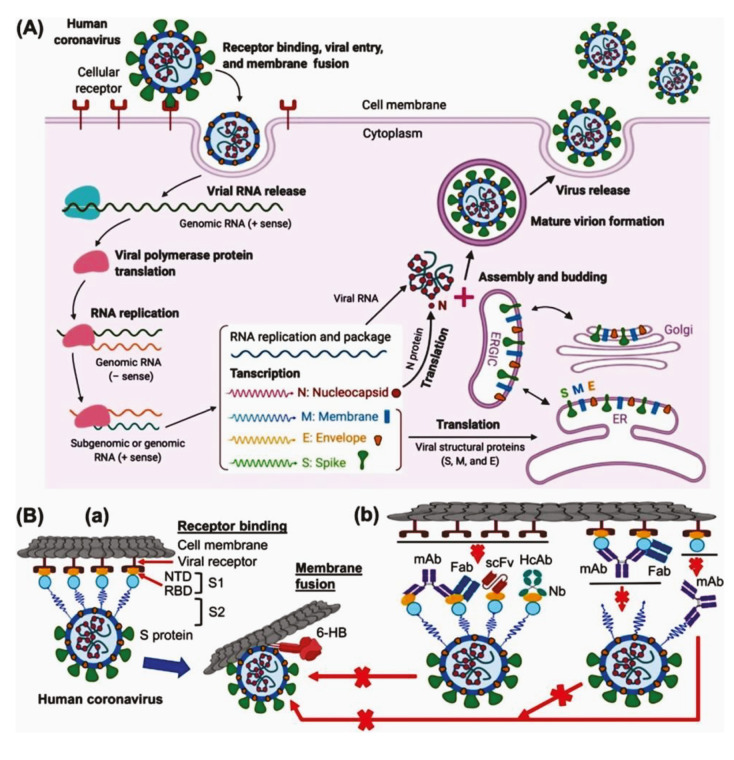
(A) Replication cycle of human coronavirus; (B) host cell receptor and viral protein (S-protein) binding and membrane fusion mechanism (Jiang et al., 2020).

Following the attachment, the cleaving of the receptor-attached spike protein and the fusion of virus into host cell are influenced by host protease. SARS coronaviruses follow 2 different paths to penetrate into the host cell based on the protease type. According to the fast path, a SARS virus enters into the cell through endocytosis at the presence of host’s pH-dependent cysteine protease cathepsin L., which leads to form a membrane bounded endosome (Figure 2A) (Simmons et al., 2013). And then, this protease induces the attached spike protein to be activated that changes the shape of endosome and helps viral envelope to fuse with the endosomal wall (Simmons et al., 2013). 

On the other hand, presence of type II transmembrane serine protease (TMPRSS2) at the outer membrane of the respiratory, urogenital and gastrointestinal epithelial cells induces proteolytic cleavage of viral spike protein. The S2’ site of the S protein comprises single lysine or arginine residues (R/K↓) which can be targeted by TMPRSS2 for cleaving, and thus, SARS and other respiratory viruses can penetrate into the host cell directly by a fusion of viral envelope and host cell membrane (Figure 2B) (Heurich et al., 2014). 

After the completion of the fusion, virus releases its nucleocapsid into the cytoplasm where the viral genome mimics the messenger RNA, and takes part in the translation process by cell ribosome (Figure 2A), and thereby, generates various replicated proteins and a replication-transcription complex (RTC) (Guo et al., 2020). By using the genomic RNA as the template, RT-complex initiates the replication of RNA and subgenomic mRNAs (Fehr and Perlmanm, 2015). The mRNAs further translates accessory proteins and structural proteins. The structural proteins (envelope glycoproteins), nucleocapsid proteins and newly synthesized genomic RNA reorganize to form progeny viruses by attributing endoplasmic reticulum and Golgi (Perrier et al., 2019). Finally, with the help of exocytose process (Figure 2A), the virion-containing viral particles discharge from the infected cell through secretory vesicles (Zhang et al., 2020b). 

### 3.2. Potentials of medicinal plants against human coronaviruses 

Without doubt, it is evident that people from different parts of the world, especially Asian countries, like India, China, Japan, Pakistan, and some parts of Africa have been utilizing plant as the herbal medicine since the prehistoric times (Hoareau and DaSilva, 1999). Medicinal plants for herbal treatment are extremely popular in rural and tribal societies, mostly due to their high scalability, which make them cheaper and affordable than modern medicine. Medicinal plants are boosted with diverse phytochemicals, such as alkaloids, terpenoids, flavonoids, phenolic acids, tannins, lignins, coumarins, stilbenes etc., which have been reported to show their potentiality against infections caused by pathogenic microorganisms (McCutcheon et al., 1995; Semple et al., 1998). 

Previous evidences have suggested that these phytochemicals may exhibit significant potential for treating viral infections; therefore, the Boots Drug Company (Nottingham, UK) showed their huge interest for the first time for developing large scale production of herbal medicine as antiviral agent, and seeking this purpose they screened at least 288 plants for estimating antiinfluenza activity (Chantrill et al., 1952). 

**Figure 3:**
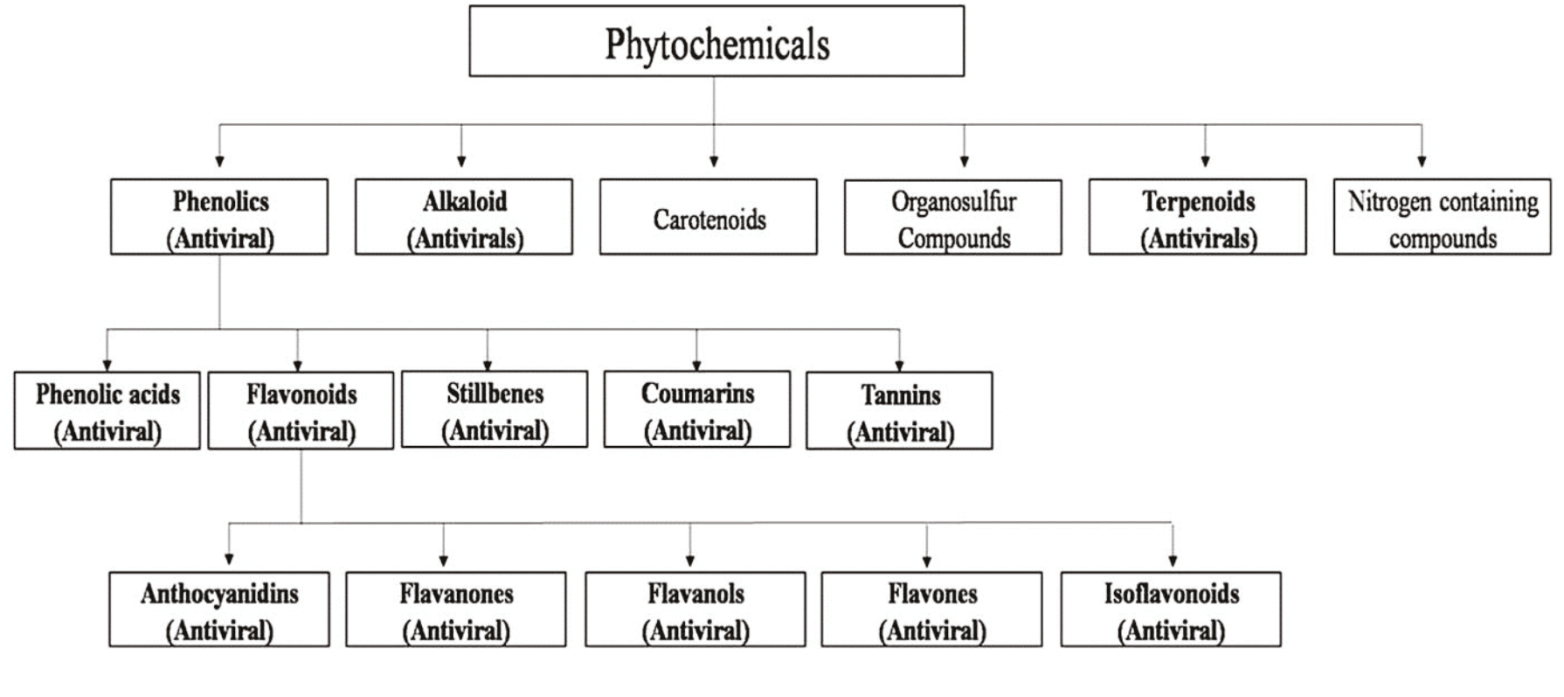
Classification of phytochemicals used as antiviral agents (Ghildiyal et al., 2020).

Moreover, different investigation on antiviral potential of medicinal plants showed that plant extracts with highly active secondary metabolites can interrupt the replication of several highly pathogenic viruses (Figure 3). Plant-based biomolecules demonstrated significant inhibitory effects on hepatitis B virus (Huang et al, 2006), the human immunodeficiency viruses (HIV) (Asres and Bucar, 2005; Lubbe et al., 2012), herpes simplex virus type 2 (Debiaggi et al., 1988) and SARS coronaviruses (Ho et al., 2007; Sassi et al., 2008; Vieira et al., 2010). 

However, this study only focuses on the antiviral potential of medicinal plants against human SARS coronaviruses since the COVID-19 pandemic by SARS-CoV-2 has become the greatest catastrophe event in the 21th century, and until now, there is no specific medication discovered to treat patients infected by this virus. Moreover, various important phytochemical groups, and their potentials for exhibiting human coronaviruses are discussed in details. 

#### 3.2.1. Essential oils 

Essential oils from many medical plants such as *Citrus* spp., *Hyssopus officinalis*, *Illicium* spp., mayweeds, tea trees, *Mentha* spp., *Santalum* spp., *Pinus* spp., thymes, ginger, and other aromatic plants with antiviral activities were well documented by several researches (Li and Peng, 2013; Akram et al., 2018; Dhama et al., 2018; Ben-Shabat et al., 2020; Wink, 2020). Essential oils are able to insert nonspecifically into the lipid double layer of the viral envelope, which alter the fluidity of membrane (Ben-Shabat et al., 2020). 

Monoterpenes, oxygenated sesquiterpenes and phenylpropanoids of essential oils are capable of disrupting the phospholipid bilayer membrane of human coronaviruses because of their lipophilic nature, which interfere the structure of viral envelope proteins during the infection (Schnitzler et al., 2003). Eucalyptol, a vital essential oil from gum trees (*Eucaliptus* spp.) was identified as an effective antiviral compound against coronavirus, especially SARS-CoV-2. This is due to the fact that this major component of eucalyptus oil consists of ether (-O), ketone (=O) and hydroxyl (-OH) groups which play the main inhibitory role against SARS-CoV-2 (Sharma, 2020). 

Resveratrol (terpenoids) can prevent the entry and post entry of MERS-CoV by suppressing viral nucleocapsid and RNA expression, which eventually inhabit viral replication. At the concentration of 125–250 μM, resveratrol were able to diminish the Vero E6 cell death (Lin et al., 2017). Moreover, jensenone, a compound obtained from eucalyptus essential oil exhibited its antiviral potential to inhabit Mpro of SARS-CoV-2 responsible for COVID-19 (Sharma and Kaur, 2020). However, further research is necessary to find the suitability of this compound as medicine for human. 

#### 3.2.2. Alkaloids

Chloroquine is a synthetic derivative of quinine, a bitter alkaloid that comes from the bark of the cinchona tree (Quina). Chloroquine is a good candidate for the development of an effective drug to treat SARS-CoV-2 because of its DNA-intercalating properties (Devaux et al., 2020). Resochin (chloroquine) is another intercalating alkaloid that was developed as a medicine, particularly for fighting malaria, which is suggested to be a potential antiviral compounds. It is believed that this compound may interrupt the replication, transcription, and translation of viral genomic material (Wink, 2020). In addition, isoquinoline alkaloids such as palmatine and chelidonine were also reported as intercalating alkaloids, suggested to be the appealing drug candidates to battle SARS-CoV-2 (Ho et al., 2019; Wing, 2020). 

In a recent study, human coronavirus (HCoV-OC43) infected MRC-5 human lung cells were utilized to estimate the antiviral activities of three pharmaceutically important bis-benzylisoquinoline alkaloids i.e. cepharanthine (CEP), fangchinoline (FAN), and tetrandrine (TET), isolated from *Stephania tetrandra* and other related species of Menispermaceae (Kim et al., 2019). These compounds also possess antiinflammatory and anticancer activities (Kim et al., 2019). However, the result showed that the replication of HCoV-OC43 inside host cells was dramatically decreased at IC50 values of 0.83, 1.01, and 0.33 μM for CEP, FAN and TET, respectively (Kim et al., 2019). Besides, expression of viral spike (S) and nucleocapsid (N) protein were also inhabited by these 3 alkaloids. In addition, a large number of alkaloids such as emetine, tylophorine and mycophenolate mofetil were reported to be very significant antiviral compounds (Shen et al., 2019; Suryanarayana and Banavath, 2020; Yang et al., 2020). 

#### 3.2.3. Phenolics, flavonoids, polyphenols, and others

Phenolic compounds, flavonoids, polyphenols, steroid, terpenoid, other active phytochemicals and their derivatives are common plant secondary metabolites that contain aromatic rings with 1 or several hydroxyl groups. The -OH groups of these compounds can form hydrogen ion bonds with positively charged amino groups of proteins, which inhibit the activity of a viral protein (Figure 1, Wink, 2020). According to Wink (2020), polyphenols are capable of binding easily with the lipoproteins of virus envelope, which can prevent the viral invasion in host cells. For a wide range of viruses, several studies confirmed the abovementioned activity of several phenolic antiviral compounds such as curcumin, luteolin-7glucoside, epicatechin gallate, catechin, demethoxycurcumin, apigenin-7 glucoside, hypericin, bavachinin, psoralidin, corylifol, mycophenolate mofetil, silvestrol and tomentin are mentioned in the Table (Ryu et al., 2010; Yu et al., 2012; Cho et al., 2013; Kim et al., 2014; Park et al., 2017; Khaerunnisa et al., 2020; Yang et al. 2020). 

Virally encoded 3C-like protease (SARS-CoV 3CLpro) is very significant viral enzyme that regulates the behaviors of coronavirus replication complexes (RTC) during viral replication (Lin et al., 2005). Therefore, this enzyme is assumed to be very crucial for the replication of SARS coronaviruses in infected host cells. Various plant-based phenolic compounds and isolated 5 major secondary metabolites from root extract of *Isatis indigotica* were used to study the antiviral potential against human corona viruses; in which, β-sitosterol, hesperetin, aloe emodin, indigo, and sinigrin were the significant phytochemicals that inhabited the cleavage activity of SARS 3CLpro enzyme with IC50 values of 1210, 365, 8.3, 752, and 217 μM, respectively (Lin et al., 2005). 

Using HPLC assay, 720 naturally occurring compounds were also applied to screen the inhibitory activity of these compounds against this enzyme (Chen et al., 2005). Among these phytochemicals, 3 naturally occurring polyphenols from Pu’er (traditional Chinese fermented tea) and black tea, i.e. theaflavin-3, 30-digallate, 3-isotheaflavin-3-gallate, and tannic acid were able to inhabit SARS-CoV 3CLpro effectively at the IC50 values of 9.5, 7, and 3 μM, respectively (Chen et al., 2005). 

Along with phenolic compounds and polyphenols, flavonoids were also found to be very effective against SARS 3CLpro enzyme of human coronaviruses. In a study, the activity of a replication protein i.e. SARS 3CLpro was prohibited by different flavor compounds available in *Litchi chinensis* seeds such as gallocatechin gallate, epigallocatechin gallate, quercetin, pectolinarin, rhoifolin, and herbacetin etc. (Gong et al., 2008). Moreover, several studies on the antiviral activity of flavonoids, especially on SARS-CoV and HCoV strains, were listed in the Table. 

Like HCoV-NL63 and SARS-CoV, SARS-CoV-2 interacts with host cell using host receptor i.e. angiotensin-converting enzyme 2 (hACE2) (Letko et al., 2020). Hence, plant-based biomolecules that are capable of preventing the interaction with this receptor could increase and strengthen the security to fight against the infection of SARS-CoV-2. Previous studies have found that several phytochemicals, such as luteolin (flavonoid) extracted from *Veronica linariifolia*, scutellarin and tetra-*O*-galloyl-β-D-glucose (TGG), polyphenolic compounds extracted from *Gallachinensis* (Yi et al., 2004; Wang et al., 2016), nicotianamine (iron chelator in plants) from soybean seeds and other food stuffs (Takahashi et al., 2015), a flavone glycoside named baicalin from *Scutellaria baicalensis* (Deng et al., 2012), emodin (quinone derivative) from genus *Polygonum* and *Rheum* (Ho et al., 2007) have striking capacity to hamper the interaction of host ACE2 and S-protein of SARS-CoV.

In an another study, molecular docking analysis was applied on different active phytochemicals including cannabinoids to determine the binding positions of these compounds with viral spike glycoprotein (S-protein) (Tallei et al., 2020). The results indicated that cannabinoids along with epigallocatechin gallate, hesperidine and pectolinarin hold remarkable binding sites, which could support them to be excellent S protein inhibitors by preventing viral attachment with host cells (Tallei et al., 2020). 

Furthermore, TSL-1 and quercetin (flavonoids) from the leaf extract of *Toona sinensis *Roem (Chen et al., 2008) as well as glycyrrhizin (saponin) from liquorice roots (Cinatlet al., 2003) and saikosaponins (triterpene saponin) (Cheng et al., 2006) evidently demonstrated strong antihuman SARS coronavirus potential by preventing the mechanism of cellular attachment, entrance, adsorption, and penetration of a virion into the host cell. 

For recognizing effective anti-SARS-CoV agents, an extensive screening through a cell-based assay was conducted on more than 10000 accessible synthetic and natural compounds and drugs (Wu et al., 2004). At nontoxic concentrations, isolated reserpine (indole alkaloids) from the extracts of *Lonicera japonica*, eucalyptus and genus *Rauwolfia*; aescin (saponins) from horse chestnut tree, and ginsenoside-Rb1 (steroids) from *Panax ginseng* exhibited excellent inhibition on the replication of SARS coronaviruses (Wu et al., 2004). 

During the viral replication, RNA-dependent RNA polymerase (RdRp) enzyme is responsible for the synthesis of both positive- and negative-strand RNA. Using swine testicular (ST) cells, plant-derived toxic substances, like cardenolides (steroid) were applied to observe the activity of antitransmissible gastroenteritis coronavirus (TGEV) activity (Yang et al., 2018). The result showed that ouabain, an important cardenolide, reduced the quantity of TGEV in a given volume of fluid at 0–3000 nM. At the inhibitory concentrations of ouabain (37 nM and 23 nM), the number of viral RNA copies was also reduced to 50% due to the suppression of viral replication and TGEV activity, which finally diminished viral yield (Yang et al., 2018).

Lectins, the carbohydrate-binding proteins from *Nicotiana tabacum*, *Allium porrum*, and *Utrica dioica* were found to be significantly potential for slowing down the viral invasion and propagation to 50% (EC50) after applying the inhibitory concentrations (less than 1.3 µg/mL) of pure extract of lectins (Yonesi and Rezazadeh, 2020). These compounds are the defense proteins in plants that can perform like immunologic receptors, and therefore could be marked for treating COVID-19 patients.

Moreover, a partial list of plants with potential antiviral phytochemicals that may possibly play a vital role to cure the COVID-19 disease used worldwide is given in the Table. Moreover, this table focused on the antiviral compounds which could be a possible drug candidate for preventing SARS-CoV or SARS-CoV-2. 

### 3.3. Clinical trials of traditional Chinese medicine (TCM) against human coronavirus 

Since there are no approved antiviral medications, drugs or vaccines, currently the main treatment strategies for treating COVID-19 patients infected by the novel SARS-CoV-2 are supportive care, a combination of wide-ranging antiviral medicines, corticosteroids, healing plasma and some antibiotics (Yang et al., 2020). Lopinavir and ritonavir are commercially available effective HIV protease inhibitors which were also utilized to treat patients with the COVID-19 in combination with IFNα-2b or with suitable antibiotics (Yang et al., 2020). This is due to the fact that ribavirin was applied widely throughout the MERS and SARS epidemic as this protease inhibitor was permitted as efficient drug for respiratory syncytial virus (RSV) infected patients (Zumla et al., 2016). On the other hand, inhibitory activity of favipiravir was observed on RNA polymerase of RNA viruses like influenza (De Clercq, 2019). Although in vitro observation established the anti-SARS-CoV-2 activity of these medications (De Clercq, 2019), a vast range of in vivo trials are required confirming their potential for battlingthe COVID-19. 

However, it has been reported that several plant species that have been used for thousands of years as TCM in controlling infectious diseases might possess anti-HCoV active compounds (Yang et al., 2020). It is very convincing that plant-based TCM showed significant positive response in preventing SARS-CoV. For instance, in a dose dependent manner, the extracts of *Ganoderma lucidum* (IC50:41.9 μg/mL), *Coriolus versicolor* (IC50:108.4 μg/mL) and *Sinomenium acutum* (IC50:198.6 μg/mL) confirmed their inhibition against SARS-CoV RNA polymerase enzyme; which interrupted viral replication (Fung et al., 2011). Using a cell proliferation assay (MTS assay), extracts from more than 200 Chinese medicinal plants were used in a study to evaluate the potential of antiviral compounds against SARS coronavirus (Li et al., 2005). Among all plants, the virus were strongly inhibited by 4 herbs namely, *Lindera aggregate*, *Pyrrosia lingua*, *Lycoris radiate,* and *Artemisia annua*, which suggested the significance of medicinal plants for the treatment of SARS associated infectious diseases. Moreover, the results of this study also confirmed that a toxic alkaloid named lycorine could be a potential agent for antiviral drug development (Li et al., 2005). 

In 2003 at the time of SARS coronavirus’ outbreak, the fatality rate until the 5th of May was more than 52% in Beijing; while about 18% in Singapore and Hong Kong. However in Beijing, death rate reduced dramatically to 1%–4% just after 10 days, which was believed to be the result of using plant-based TCM as a supplement medication with conventional therapy (Chen and Nakamura, 2004). Therefore in China, after gathering knowledge from the treatment strategies of MERS and SARS patients as well as following the existing Chinese clinical guideline, TCM have been incorporated with conventional medicine to treat the patients infected by SARS-CoV-2 (Yang et al., 2020). 

Two traditional Chinese herbal medicines ‘*Sang Ju Yin’ *and ‘*Yu Ping Feng San*’ were utilized to observe the changes of defense system in host cell, and the result showed that it may possibly alter T cells which help to boost up the host defense capability (Poon et al. 2006). In an another independent study, the supplementary treatment with traditional Chinese herbal medicines confirmed remarkable effects in symptom development, which eventually help to shorten disease course caused by viral infection (Hsu et al., 2006). Moreover, these positive outcomes of traditional Chinese medicines in this controlled clinical trial were also supported by laboratory based in vitro studies. 

**Figure 4:**
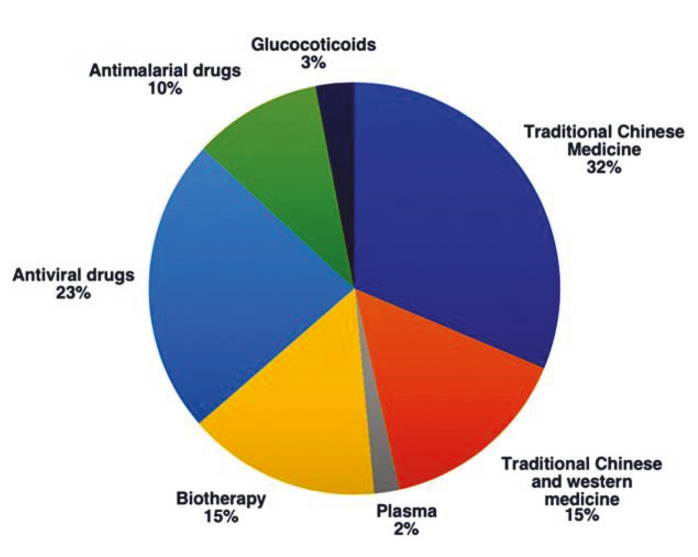
Percentage of the tested experimental drugs registered in China for 2019-nCoV therapy (Zhang et al., 2020a).

A comparative study was performed to measure the application rate of different experimental drugs registered in China, based on the current treatment strategies as well as considering recent published literatures on COVID-19 pandemic (Figure 4). Among all these experimental drugs, such as western medicine like antimalarial drugs, antiviral drugs and biotherapy, traditional Chinese medicines, application of glucocorticoids and plasma; TCM has the highest rate with 32% (Zhang et al., 2020a). Therefore, considering the homology of SARS-CoV-2 and SARS-CoV, all of the abovementioned studies on TCM may shed light on the production of natural compound escapable of inhibiting SARS-CoV-2. 

## 4. Concluding remarks 

Less toxic natural products from traditional medicinal plants with antioxidant and antimicrobial properties have been frequently utilized for thousands of years for treating various infections due to their minimal side effects. As a result of irrepressible outbreak of COVID-19 and unavailability of approved medications, complementary and substitute treatments using plant-based phytochemicals could be incredibly promising for managing this pandemic by reducing the severity of infection caused by SARS-CoV-2. This is due to the fact that various phytochemicals such as lycorine, hypericin, emodin, tylophorine, ouabain, mycophenolate mofetil, silvestrol, myricetin, scutellarein etc. exhibited strong inhibitory effects against SARS-CoV-2 and other human coronaviruses. Besides, plant-based traditional Chinese medicines as supplementary medication showed their potential for reducing the fatality rate during the outbreak of SARS-CoV. However, vast range of double-blinded clinical studies with strict protocols is required to estimate the accurate potential of antiviral phytochemicals against COVID-19 with the aim of ensuring the fulfillment of international acceptable standards.
